# Dreams and Trauma Changes in the Manifest Dreams in Psychoanalytic Treatments – A Psychoanalytic Outcome Measure

**DOI:** 10.3389/fpsyg.2021.678440

**Published:** 2021-09-14

**Authors:** Tamara Fischmann, Gilles Ambresin, Marianne Leuzinger-Bohleber

**Affiliations:** ^1^International Psychoanalytic University Berlin, Berlin, Germany; ^2^CHUV, Lausanne, Switzerland; ^3^Universitätsmedizin der Johannes Gutenberg-Universität Mainz, Mainz, Germany

**Keywords:** dreams, memory reconsolidating, research methods, psychotherapeutic (psychoanalytic) treatment, differential psychotherapy research

## Abstract

Although psychoanalysts are interested in symptom reduction as an outcome, they are looking for instruments to measure sustaining changes in the unconscious mental functioning. In this article it is discussed that conceptually well-founded transformation of manifest dreams analyzed with precise empirical methods could be considered as a promising indicator for such therapeutic changes. We are summarizing a dream generation model by Moser and von Zeppelin which has integrated a large interdisciplinary knowledge base of contemporary dream and sleep research. Based on this model the authors have developed a valid and reliable coding system for analyzing manifest dreams, the Zurich Dream Process Coding System (ZDPCS). One exemplary dream from the beginning and one from the third year of a severely traumatized, chronic depressed patient from the LAC Depression Study collected in psychoanalytic sessions as well as in the sleep laboratory have been analyzed applying the ZDPCS. Authors hypothesize that transformation in dreams as measured with the ZDPCS is the result of memory processes of traumatic embodied memories in the state of dreaming.

## Introduction

Both the clinical practice of psychoanalysis and its extra-clinical research have been enriched and inspired, but also challenged, by various interdisciplinary dialogues in recent decades. To mention just one example: it was long held in psychoanalysis that, following Sigmund Freud’s dream theory [1900 (1961)], it was the analyst’s task to investigate the latent meaning of a dream by systematically exploring the associations to a patient’s dream during an analysis session. As will be discussed in the following article, both experimental dream research and dialogue with neurobiological memory research have critically challenged this limitation and have opened new doors to the systematic exploration of the manifest dream. As will be briefly illustrated by an example of a patient from the LAC study (Long-term Analysis of Chronic depression; Leuzinger-Bohleber et al., 2019a,b), this opens up, among other things, the fascinating possibility of systematically investigating the changes of (manifest) dreams in psychoanalyses as indicators of the transformations of the unconscious micro-worlds (Moser and Hortig, 2019). If systematic changes can be detected in dreams, they would provide a measure more akin to the mental (unconscious) transformations focused on in psychoanalysis than some criteria accepted in times of evidence-based-medicine, for comparative psychotherapy studies (almost predominantly self and other assessments of symptom reduction).

As we will discuss, we therefore see these interdisciplinary dialogues as an opportunity for genuine psychoanalytic outcome research. In a detailed article, we have discussed the contribution of neurobiological memory research by the research group of Lane et al. (2015), especially their concept of memory reconsolidation, to this concern (cf. Leuzinger-Bohleber et al., 2020). We also referred to previous work on the modification of manifest dreams in psychoanalyses in a former psychotherapy outcome study (by Leuzinger-Bohleber, 1989 replicated by Kächele et al. (2015), see also Kamp et al., 2019; Pap, 2021). In the frame of this short article, we limit ourselves to the presentation of a dream generating model developed by Ulrich Moser’s psychoanalytic research group in Zurich. The authors have attempted to integrate the current state of sleep and dream research into their model. What is relevant for psychotherapy research is that based on this model they have developed a sophisticated, valid and reliable system for the investigation of manifest dream content, the Zurich Dream Process Coding System (ZDPCS). We hypothesize that the ZDPCS provides an instrument that may foster the emerging role of interdisciplinary research in clinical psychoanalysis. Using two dreams of an analysand from the LAC study, it is illustrated that this coding system is suitable to capture relevant changes during a psychoanalysis.

### Neuropsychoanalytic Dream Research

Dreams are at the foundation of psychoanalysis ever since Freud (1961) claimed them to be the royal road to the unconscious. Moreover, as we humans spend about one-fifth of our sleeping time dreaming, it may well be assumed that it fulfils a multitude of biological and cognitive/affective roles in humans as, e.g., a possibility to digest relevant information and unsolved conflicts by means of mental processes during sleep (see e.g., Cartwright et al., 2006; Zhao et al., 2018; Siclari et al., 2020).

### Dreams as Guardians of Sleep and Memory Processes

The first scientific hypothesis regarding the function of dreaming was that of Freud (1961) who proposed that dreams were the “guardians of sleep.” In his view, sleep is characterized by endogenous stimuli that activate wishes (or vice versa), which would evoke motor activity while awake, thus threatening to disrupt sleep. That is why dreams are generated: for the purpose to “divert” potentially sleep-disturbing wishes via hallucinatory wish-fulfillment from otherwise necessary motor activity. “Recent sleep research has convincingly demonstrated that sleep is accompanied by potentially disturbing endogenous arousal and motivational events, disturbed by surges of midbrain dopaminergic activation in the mesocortical-mesolimbic circuit” (Fischmann and Leuzinger-Bohleber, 2018, p. 140; for therapeutic consequences^1^, see e.g., Leuzinger-Bohleber, 2015b). The latter being “the most vigorous exploratory search activity an animal is capable of” (Panksepp, 1998, p. 145). This raises the question than why do we remain asleep? According to Freud, we do so by dreaming (Freud, 1961). But dreaming’s purpose is not alone to guard sleep, it is also theorized to play a major role in memory processing during sleep. An interesting hypothesis has been put forth in recent years that sleep and dreaming contribute to and influence memory consolidation and re-consolidation in a significant manner (Zhao et al., 2018; see also Lane et al., 2015).

Rapid Eye-Movement (REM) is a component of sleep, which is characterized by cyclical arousal states and where limbic forebrain structures are activated together with the amygdala, while the hippocampus is inhibited (Stickgold et al., 2001). In light of this activation process, it can be assumed that instead of reactivation of episodic memories a dream arises during this sleep phase. This provides evidence for the role of dreaming in memory consolidation, where dreams are thought to be constructed primarily from weak neocortical associations available during REM sleep (cf. Siegel, 2001; Diekelmann et al., 2009; Scarpelli et al., 2019). It is further likely that, as a result, the brain attempts to recognize and evaluate these resulting novel cortical associations in the context of their accompanying emotions mediated by limbic structures, giving dreams their typically unpredictable, bizarre, and emotionally charged character (Stickgold et al., 2001). Due to the relative loss of motor function in REM sleep, the sleeping individual is forced to resort to pictorial imagination to achieve arousal discharge. The latter is a compensation that can be assumed to be a functional consequence of REM dreaming, in addition to the accompanying strengthening or weakening of the specifically activated associations. This latter link seems to be confirmed by increased dreaming of tetraplegic patients due to Guillain-Barré syndrome and similar lower-motor-neuron lesions (Cochen et al., 2005).

As for the function of dreaming with respect to memory processes there are several theories that propose for example that the emergence of memories in dreams reactivates those memory traces in their original (perception-like) states, thus promoting learning while dreaming. It has also been shown that the embedding of emotionally relevant memory elements strengthens and consolidates them. It is also known that dreaming about newly learned material improves later recall of this material (for reviews see Payne and Nadel, 2004; Nielsen and Stenstrom, 2005).

## The “Dream-Generation-Model” by Moser and Von Zeppelin – An Attempt to Integrate Psychoanalytic and Interdisciplinary Knowledge on Dreams^2^

Moser and von Zeppelin (1996) consider the sleep dream as a simulated micro-world. The simulation is driven by affectivity, leading in the end to images of entities involved (*subject, object*, and *things*) and relationships linking them. A dream is triggered by events of the previous day or night. This event reactivates unresolved conflicts and problems (*current concern*). The dream has the function of retrospective problem solving. While in the waking state, in contrast to the dream state, we react immediately to our environment and by that consolidate information into our memory, there is often a restriction of consolidation processes in the waking state due to capacity restrictions of the memory system. It is interesting to note, that these consolidation processes^3^ also take place during sleep in a so-called “off-line” mode. This is how new information is integrated into long-term memory. As the dream is looking for a solution of reactivated conflicts and problems the action and expression components of the affects are inhibited in the dream state as representation of the inner life dominates. But the range of affect modulation is significantly larger than in the micro-worlds of the waking state and stress is absorbed via imagination and via cognition. Affects may nevertheless become too strong and will lead to *interrupts* of the dream and might even cause waking-up, so that the dream microworld contains *situation sequences* (Sit) with *interrupts*. The dream is not involved in regulating concrete-real object relationships, but rather works with memories and with acquired solution and defense strategies [called *self-* and *object-models* and generalized interaction-representations (*RIGs*)^4^, or, on a different level of observation, rather prototypical affective microprocesses (PAMs)]^5^. According to this dream-generation-model, dreams often start with a *positioning field* (PF) without interactions. What appears in this PF is regulated by a security principle, which prevents the emergence of threatening affects by means of distance relations. Once the affective cathexis of the microworld is very strong, the dream narrative initiates an *interactive field of interactions* (IAF), where the PF is still there “by default” as a background presence. The dream contains procedures of approaching and distancing from the intended wish fulfillment (i.e., problem solving) via regulation of involvement and commitment as well as via interactive procedures of shaping its security regulation. Via a feedback-loop (reentry), the dream may be interrupted, if the affectivity gets too unbearable, and a new PF is created, thus increasing safety for the next situation. Every dream sequence contains a PF, which includes, always per situation, all mentioned elements: subject, objects, inanimate things, often summarized in a *PLACE*, which is a kind of spatial micro-world (for short overview of the abbreviations, see Supplementary Material).

**FIGURE 1:**
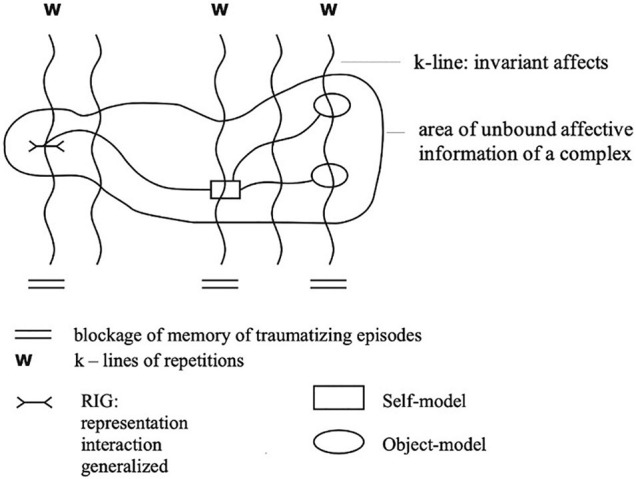
Memory Model of conflictual complexes adapted from Moser and von Zeppelin (1996). W, repetitions (i.e., Wiederholungen) caused by invariant affects, which are not yet bound in the personal-historical life narrative of the individual; areas of unbound affective information: complexes consisting of self- and object-models and generalized interaction representation (RIG), which are grouped by k-lines of invariant affects, causing blockage of memory of closely related traumatizing episodes.

Within the micro-world dream, which is considered to be an affective-cognitive bundle, initiated by current concerns, a “dream complex” can be seen as a template that enables the dream to be organized accordingly. Thus, a “dream complex” can be assumed to consist of one or more complexes that have their origin in conflictual and/or traumatic experiences stored in non-declarative long-term memory. These complexes have ultimately found their condensates in introjects, i.e., affective-cognitive templates of conflictual or traumatizing memory traces. When these introjects are triggered by closely related current concerns from the outside, these “dream complexes” may be considered structurally like stored situations of the introjected complex, and a dream emerges in search for resolving the complex. The memory traces of such complexes are characterized by invariant intense, unbearable affects connected by so-called k-lines and are stored isolated from memories with relational reality. Each of these isolated complexes contain unbound affective information and represent links between self- and object-models and generalized interaction-representations (PAMs), which are accompanied by convictions and a hope for wish-fulfillment (i.e., problem-solving). They have a repetitive character (W), as they are in constant search for a solution in order to get rid of the disturbing unbound affects^6^. We cannot describe this elaborated model of dream generation and the coding system based on it in more detail here but hope that the following illustration (see Figure 1) may elucidate this model (An English publication of the model is in preparation).

Outside the dream world, where the reality principle prevails, these conflictual or traumatic complexes cannot be thought of declaratively as they are being pushed into the unconscious^7^ because of their intolerability. In the dream world, in which the pleasure principle prevails, the affective information comes more easily to the fore, and the “dream organizer” (i.e., the dreaming sleeper) seeks a solution by creating a tolerable micro-world in which the affective information suppressed or dissociated in the waking state can come “alive” and become solvable (cf. Figure 1)^8^. The “dreamlike” problem solution of such unbearable complexes is facilitated by balancing innate tendencies for security and the desire for involvement. Whenever this fails in a dream, the dream scene is interrupted and either a new one is created, or the dreamer wakes up in a state of panic. Thus, the number of interrupts of dream scenes within a dream may, according to our first empirical findings (Fischmann et al., 2013; Fischmann and Leuzinger-Bohleber, 2018), be considered as one indicator for change. The less interrupts within a long dream the closer to the solution of the dream complex the dreamer is. Of course, in shorter dreams we find less interrupts: these dreams break off early.

Based on this model of dream-generation Moser and von Zeppelin (1996) have developed a coding system which can be used to analyze the manifest dream content – the Zurich Dream Process Coding System (ZDPCS). Inter-rater reliability based on 20 dreams taken from psychotherapies have been reported for the coding-system to be very satisfactory κ (Cohen’s kappa) = 0.936 (Döll-Hentschker, 2008, p. 238). It can be applied to investigate systematic changes in the manifest dream content for instance of dreams of analysands during their psychoanalyses, as was done in the LAC depression study. In an attempt to further validate this instrument (ZDPCS), REM dreams elicited in a sleep laboratory of some of the analysands, who have agreed to have their severe sleep disturbances examined there, were investigated and compared to their “clinical” dreams reported during psychoanalytical sessions with very interesting findings (see Fischmann and Leuzinger-Bohleber, 2018 and Supplementary Material). Even though the dream content differed, the structure of the laboratory dream and the dream reported during sessions was identical – a finding which was important for the systematic investigation of the changes in the manifest dreams during the long-term treatments (see Supplementary Material for the specific steps taken to apply the ZDPCS).

In their newest publication Moser and Hortig (2019) slightly revised and modified the ZDPCS and present the conceptualization as well as the different categories of the coding system in great detail. In the last part of their book (part VI) they discuss different interpretations of dreams based on the ZDPCS as well as a dream series of the same patient which we are presenting in this article^9^.

## Case Illustration

More than 80% of the 252 patients examined in the LAC Study, suffered from severe childhood trauma (see Negele et al., 2015). A salient feature of traumatic events, according to psychoanalytic trauma theories is that an individual is suddenly and unexpectedly confronted with an extreme situation of utter helplessness and impotence in relation to extreme pain and threat to life without help from others, thus losing a basic sense of self-agency. Therefore, in a traumatic situation, the basic trust in a helping “other” and an active self is destroyed, which has sustaining consequences (see e.g., Bohleber, 2010; Leuzinger-Bohleber, 2015b).

Exactly such unbearable, traumatic situations characterized the nightmares of the patient X. from the LAC study and his dreams of the first 6 months of psychoanalysis: The dream-self is captured in an extremely dangerous, live threatening situation. He is flooded with panic and anxiety and has lost any capability to liberate himself from this situation (see e.g., Varvin et al., 2012). Here just one example from a dream of Mr. X. in one of the sessions during the beginning phase of his psychoanalysis.

*“A nightmare: I am in a narrow tunnel, kind of a tube. Behind me my brother is crawling. We cannot go backward – behind us it the stormy sea. The tunnel turns to be more and more narrow. I am waking up in panic.^10^”*

In the frame of this article, we can only illustrate the analyses of two exemplary dreams of this patient (a) from the first 6 months of psychoanalysis and (b) from the third year of psychoanalysis (cf. Table 1):

**TABLE 1:** ZDPCS dream coding a clinical dream of the first 6 months of psychoanalysis.

**Dream narrative**	**Sit**	**PF**	**LTM**	**IAF**
**Dream from clinical situation**				
I am in a narrow tunnel, kind of a tube	S1	SP (Dreamer) PLACE (tunnel) ATTR (narrow) ATTR (tube)		
Behind me my brother is crawling	S2	SP (Dreamer) OP_1_ BEK (brother) POS REL	LTM	
We cannot go backward – behind us is the stormy sea	S3	SP (Dreamer) OP_1_ BEK (brother) PLACE (sea) ATTR (stormy) POS REL		IR.C RES LTM FAIL (cannot go backward)
The tunnel becomes narrower and narrower	S4	SP (Dreamer) PLACE (tunnel) ATTR (narrow)		IR.D (IR.S)
I wake up in panic	EX AFF- R			

*Sit, situation; PF, positioning field; LTM, loco-time motion, IAF, interaction field. See Supplementary Material for abbreviations.*

As is discussed in detail in another article (see Fischmann and Leuzinger-Bohleber, 2018; Leuzinger-Bohleber, 2018) the manifest dreams of this severely traumatized patient changed obviously during psychoanalysis. Here an example of a clinical dream of this patient in the third year of psychoanalysis:

*“I played with the famous jazz guitarist Ralf Towner. It went quite well, and it was fun. I didn’t fail and the neck of the guitar was not soft^11^ (laughs). The guitarist played along with my improvisations and held back. Of course, I knew that he is better than me, but this did not matter – it was just great fun*…*”* (Fischmann and Leuzinger-Bohleber, 2018, p. 149).

From the content of the manifest dream in the third year of his psychoanalysis it becomes evident that he is highly affectively aroused and is awakened by it (see Table 2), like in the dream from the beginning of his psychoanalysis (cf. Table 1), but now he is joyfully excited, and he is able to interact responsively with a “helping” object during most of the dream (cf. Table 2).

**TABLE 2:** ZDPCS dream coding comparing clinical dream of the third year of psychoanalysis (Fischmann and Leuzinger-Bohleber, 2018, p. 153).

**Dream narrative**	**Sit**	**PF**	**LTM**	**IAF**
**Dream from clinical situation**				
I play with the famous jazz guitarist Ralf Towner. It goes quite well	S1	SP (Dreamer) OP BEK (Towner) ATTR (famous) ATTR (jazz g.)		IR. C RES
It is fun	EX AFF R			
I don’t fail and the neck of the guitar is not soft	S2	SP (Dreamer) CEU (guitar) PART OF (neck) ATTR (not soft)		
The guitarist plays along with my improvisations and holds back.	S3	SP (Dreamer) OP BEK (guitarist) ATTR (held back)		IR.C RES
Of course, I know that he is better than me, but this does not matter	C.P.			
It is just great fun	EX AFF R			

*Sit, situation; PF, positioning field; LTM, loco-time motion; IAF, interaction field. See Supplementary Material for abbreviations.*

We hope to have illustrated to a degree by these two exemplary dreams how Mr. X’s early traumatization has become observable in his manifest dreams and how this changed during the treatment. The underlying traumatic complex, of growing up with an abusive, alcoholic mother and an absent father, governed the dream organization at the beginning of treatment exhibiting his panic and anxiety and incapability of freeing himself from an extremely dangerous situation. This changed during the treatment where the traumatic complex was successively better integrated in the psychic functioning of the patient. The ZDPCS coding revealed how he established an increasing feeling of self-agency, control and basic trust in a helping “other.” We interpret these findings that the “embodied memories” of the traumatization were successively better integrated in the psychic functioning of the patient (e.g., connected with this just mentioned increasing feeling of self-agency, control and basic trust in a helping other in the dream plots).

Mr. X. belongs to the selected patients of the LAC Study who was willing also to be investigated in the dream laboratory. This enabled us, as shortly mentioned above, to look at dreams he reported in the clinical situation and compare them to dreams of the same week elicited at the sleep laboratory using the ZDPCS method. We wanted to study whether we could discern changes of dreams like those discussed in the paragraph above. We were also interested to see if those changes occurred in both types of dreams – the laboratory dreams and the ones reported in the clinical situation. As we discuss in the Supplementary Material, we indeed were able to show that there are similar changes in the dream contents in the clinical as well as in the laboratory dreams (see Supplementary Material).

## Summary and Discussion

Changes in psychotherapeutic treatments are usually measured by the amount of symptom reduction. Although psychoanalysts are interested in this as well as a general outcome measure, they are above that looking for sustaining changes in the unconscious mental functioning. One way of achieving this is by looking at changes in manifest dream content using a precise empirical method as a promising indicator of therapeutic change as is suggested here in this article.

In the beginning of therapy, patients repeatedly report frequent nightmares which are often connected to (early) traumatization of the analysands. Nightmares are triggered not only by an extreme overwhelming anxiety, but also by the feeling of a missing, holding, containing other. This is well known from trauma theory, where trauma is defined as a situation in which the basic trust in a helping “Other” and self-agency is destroyed—an experience with sustaining consequences (see e.g., Bohleber, 2010). In their model of the generation of dreams, Moser and von Zeppelin (1996) propose that traumatic complexes, stored in non-declarative long-term memory, can be characterized by the fact that extreme affects are not integrated (bonded) in a structure of human relationships. When a solution for the traumatic complexes is searched for in dreams the dream-subject tries to find a way out of the traumatic situation of extreme helplessness, impotence and unbearable negative affects, such as panic, despair, rage and death anxiety, by trying to gain a more active stance and control over the dangerous situation. This is done by creating dream situations over and over again, governed by the wish for involvement (self-agency) and the need for security. In combination with a successful psychoanalysis the dream-subject successively gains a more active stance, which can also be seen in his dreams. This marks a “turning point” in the psychoanalytic process. The case illustration given here can only hint, but not systematically show or theoretically discuss in detail, the successful achievement of how analysis brought the (split–off) trauma with its unbearable affects and unconscious beliefs back into the psychoanalytic relationship. This is postulated to have led to a modification of the patients unconscious convictions that “no one-but no one” is interested in me when I am in an unbearable, life threatening situation with complete helplessness and impotence, without any self-agency. It is well understood that the traumatic experience – and the memory of it – cannot be extinguished by the experience in the transference/countertransference of the psychoanalytic relationship but may lose its quality of the unbearable horror as well as the psychic quality of nightmares will.

Such changes in the quality of unbearable horror from which patients suffer are indicative of positive changes in psychotherapies. They are difficult to capture because they must be held unconscious by defenses to make them reasonably bearable. However, as we have tried to show here, they can be made visible through the analysis of dreams, both the latent contents of dreams in the psychoanalytic situation and manifest dreams elicited in an experimental as well as in therapeutic situations. Dreams are key to understanding unconscious conflicts and fantasies and can provide clues to possible transformations in psychic functioning. Often such transformations take place in a hidden way. Nevertheless, the psychoanalytic investigation of changes in long-term psychoanalytic therapies by means of a systematic clinical and extra-clinical approach of changes in dreams offers the possibility to capture such changes beyond symptom reduction in a clinically relevant way (see also Leuzinger-Bohleber, 2015b; Leuzinger-Bohleber and Fischmann, 2018). We thus hope to have made a valuable contribution to such comparative outcome studies. Another hope is that we could show that a dialogue between psychoanalysis and the neurosciences is fruitful in comparative psychotherapy research (see Fischmann et al., in preparation).

## Data Availability Statement

The raw data supporting the conclusions of this article will be made available by the authors, without undue reservation.

## Ethics Statement

The studies involving human participants were reviewed and approved by the Ethik-Kommission bei der Landesärztekammer Hessen. The patients/participants provided their written informed consent to participate in this study.

## Author Contributions

TF and ML-B contributed to conception of the manuscript. TF organized the database and performed the statistical analysis. GA, TF, and ML-B wrote the sections of the manuscript. All authors contributed to manuscript revision, read, and approved the submitted version.

## Conflict of Interest

The authors declare that the research was conducted in the absence of any commercial or financial relationships that could be construed as a potential conflict of interest.

## Publisher’s Note

All claims expressed in this article are solely those of the authors and do not necessarily represent those of their affiliated organizations, or those of the publisher, the editors and the reviewers. Any product that may be evaluated in this article, or claim that may be made by its manufacturer, is not guaranteed or endorsed by the publisher.

## Footnotes

1 Clinical and interdisciplinary knowledge about the emergence of dreams and their possible transformations during treatments is also highly relevant for the practicing psychoanalyst, as we have discussed in various publications (e.g., in Leuzinger-Bohleber, 2015a,b). To briefly mention just one example here: Some of the chronically depressed analysands conveyed to the analyst for a long time their deep, unconscious conviction that the depression would resist any change, a conviction that of course had to be understood in detail in the analytic work and worked through therapeutically.

2 This section is based in a former publication by Fischmann and Leuzinger-Bohleber (2018) and Leuzinger-Bohleber et al. (2020).

3 It is well established that reconsolidation requires a night of sleep (see Leuzinger-Bohleber et al., 2020).

4 Stern (2020) and his research group have developed the concept of RIGs (Representation Interaction Generalized) which had a great influence on empirical infant research. It is connected to the concept of schema or generalized cognitive-affective patterns which have been developed in the central early relationships and which determine mostly unconsciously the expectations to current relationships in the real world or in the transference.

5 Hortig and Moser (2012) rather speak in this later work of prototypical affective microprocesses (PAMs), which are defined as dyad-specific processes in affective relationship regulation (see also Bänninger-Huber, 1992). They are a product of both participants. In the case of mismatch, dysfunctional PAM structures are formed.

6 Another way of saying this – referring to the terminology of attachment research – is as follows: An internal working model (IWM) of the social world is created in childhood. In the case of abuse/neglect, the IWM is well adapted to the abusive context, but then is maladaptive later in life outside the family of origin. The IWM generates predictions that don’t conform to the social reality. Thus, there are major prediction errors. These prediction errors result in affective responses. They are “unbound” in the sense of being activated and not resolved via an updated prediction or a change in circumstances/sensory input that conform to prediction. Perhaps the function of dreaming is to rework the RIG or IWM to minimize future prediction error.

7 Lane comments that the emotional responses cannot be mentalized or mentally represented. They are not repressed. Dissociation can lead to lack of mental representation. I would also say that they are not “pushed into the unconscious” but rather that automatic emotional responses are too intense to be constructed as discrete, specific conceptualized experiences. In other words, they never made it into conscious awareness as discrete experiences.

8 In other words, a function of dreaming is to establish a basis for the affect to become attached to meaning systems and become mentalizable. It could also be the case that this function during dreaming contributes to the encoding of memories along emotional lines such that in the future memories associated with a given emotional state are more easily accessible when in that emotional state.

9 In our analyses we used an older version of the ZDPCS.

10 In another article, we have compared these clinical dreams with dreams of the same patients during the same sequence of his psychoanalyses which he had reported in the sleeping laboratory (see Fischmann and Leuzinger-Bohleber, 2018). Moser and Hortig (2019) have analyzed the same dream in their revised and modified coding-system (p. 200 ff.).

11 The patient refers to another “funny” dream. Before the dream he had a conflict with his wife which wasn’t treated openly. Instead, the conflict led to an erectile dysfunction. Then he dreamed, that he played on a guitar which had a very soft neck.

## References

[B1] Bänninger-Huber E. (1992). Prototypical affective microsequences in psychotherapeutic interaction. *Psychother. Res.* 2 291–306. 10.1080/10503309212331333044

[B2] Bohleber W. (2010). *Destructiveness, Intersubjectivity, and Trauma: The Identity Crisis of Modern Psychoanalysis.* London: Karnac Books.

[B3] Cartwright R. Agargun M. Y. Krikby J. Friedman J. K. (2006). Relations of dreams to aking concerns. *Psychiatry Res.* 141 261–270. 16497389 10.1016/j.psychres.2005.05.013

[B4] Cochen V. Arnulf I. Demeret S. Neulat M. L. Gourlet V. Drouot X. (2005). Vivid dreams, hallucinations, psychosis and REM sleep in Guillain–Barré syndrome. *Brain* 128 2535–2545. 10.1093/brain/awh585 16000335

[B5] Diekelmann S. Wilhelm I. Born J. (2009). The whats and whens of sleep-dependent memory consolidation. *Sleep Med. Rev.* 13 309–321. 10.1016/j.smrv.2008.08.002 19251443

[B6] Döll-Hentschker S. (2008). *Die Veränderung von Träumen in Psychoanalytischen Behandlungen. Affekttheorie, Affektregulierung und Traumkodierung [Changes in Dreams in Psychoanalytic Treatments. Affect Theory, Affect Regulation, and the Coding of Dreams].* Frankfurt am Main: Brandes & Apsel.

[B7] Fischmann T. Ambresin G. Leuzinger-Bohleber M. (in preparation). Dream series during psychoanalyses with chronic depressed, early traumatized patients in the frame of the MODE Study.

[B8] Fischmann T. Leuzinger-Bohleber M. (2018). “Dreams,” in *Neuropsychodynamic Psychiatry*, eds Böker H. Hartwich P. Nordhoff D. (New York, NY: Springer), 137–215.

[B9] Fischmann T. Russ M. O. Leuzinger-Bohleber M. (2013). Trauma, dream, and psychic change in psychoanalyses: a dialog between psychoanalysis and the neurosciences. *Front. Hum. Neurosci.* 7:877. 10.3389/fnhum.2013.00877 24381554 PMC3865430

[B10] Freud S. (1961). “The interpretation of dreams,” in *Standard Edition of the Complete Psychological Works of Sigmund Freud*, Vol. 4–5 ed. Strachey J. (London: Hogarth Press). (Original work published 1900).

[B11] Hortig V. Moser U. (2012). Interferenzen neurotischer Prozesse und introjektiver Beziehungsmuster im Traum. *Psyche* 66 889–916.

[B12] Kächele H. Albani C. Pokorny D. (2015). “From a psychoanalytic narrative case study to quantitative single-case research,” in *Psychotherapy Research – Foundations, Process and Outcome*, eds Gelo O. C. G. Pritz A. Riegen B. (Wien: Springer-Verlag), 367–379. 10.1007/978-3-7091-1382-0_19

[B13] Kamp G. Lackinger F. Löffler-Staska H. (2019). Therapieerfolgsindikatoren in Träumen. Verbale relationen und bekannte personen in Träumen nach trauma mit und ohne posttraumatischer belastungsstörung. *Psychopraxis Neuropraxis* 22 176–179. 10.1007/s00739-019-0573-8

[B14] Lane R. Ryan L. Nadel L. Greenberg L. (2015). Memory reconsolidation, emotional arousal and the process of change in psychotherapy: new insights from brain science. *Behav. Brain Sci.* 38 1–19. 10.1017/S0140525X14000041 24827452

[B15] Leuzinger-Bohleber M. (1989). *Veränderung Kognitiver Prozesse in Psychoanalysen. Bd. 2: Fünf aggregierte Einzelfallstudien.* Berlin: Springer (PSZ).

[B16] Leuzinger-Bohleber M. (2015a). *Finding the Body in the Mind – Embodied Memories, Trauma, and Depression.* London: International Psychoanalytical Association.

[B17] Leuzinger-Bohleber M. (2015b). Working with severely traumatized, chronically depressed analysands. *Int. J. Psychoanal.* 96 611–636. 10.1111/1745-8315.12238 26173883

[B18] Leuzinger-Bohleber M. (2018). “Concepts of empirical and clinical research in psychoanalysis and neuropsychoanalysis,” in *Neuropsychodynamic Psychiatry*, eds Böker H. Hartwich P. Nordhoff D. (New York, NY: Springer), 563–578. 10.1007/978-3-319-75112-2_28

[B19] Leuzinger-Bohleber M. Ambresin G. Lane R. D. Fischmann T. (2020). Changes in the manifest dreams an outcome indicator of psychoanalytic treatments? Informed by research on dreams, trauma, memory reconsolidation. *Neuropsychoanalysis: an Interdisciplinary Journal for Psychoanalysis and the Neurosciences*

[B20] Leuzinger-Bohleber M. Fischmann T. (2018). “Neuroscientifically inspired psychoanalysis. Chronic depression as a paradigmatic example,” in *Neuropsychodynamic Psychiatry*, eds Böker H. Hartwich P. Nordhoff D. (New York, NY: Springer), 579–597. 10.1007/978-3-319-75112-2_29

[B21] Leuzinger-Bohleber M. Hautzinger M. Fiedler G. Keller W. Bahrke U. Kallenbach L. (2019a). Outcome of psychoanalytic and cognitive-behavioural long-term therapy with chronically depressed patients: a controlled trial with preferential and randomized allocation. *Can. J. Psychiatry* 64 47–58. 10.1177/0706743718780340 30384775 PMC6364135

[B22] Leuzinger-Bohleber M. Kaufhold J. Kallenbach L. Negele A. Ernst M. Keller W. (2019b). How to measure sustained psychic transformations in long-term treatments of chronically depressed patients: symptomatic and structural changes in the LAC Depression Study of the outcome of cognitive-behavioural and psychoanalytic long-term treatments. *Int. J. Psychoanal.* 100 99–127. 10.1080/00207578.2018.1533377 33945717

[B23] Moser U. Hortig V. (2019). *Mikrowelt Traum. Affektregulierung und Reflexion.* Frankfurt am Main: Brandes & Apsel.

[B24] Moser U. von Zeppelin I. (1996). *Der Geträumte Traum. Wie Träume Entstehen und Sich Verändern.* Stuttgart: Kohlhammer.

[B25] Negele A. Kaufhold J. Kallenbach L. Leuzinger-Bohleber M. (2015). Childhood trauma and its relation to chronic depression in adulthood. *Depress. Res. Treat.* 2015 650804–650811. 10.1155/2015/650804 26693349 PMC4677006

[B26] Nielsen T. A. Stenstrom P. (2005). What are the memory sources of dreaming? *Nature* 437 1286–1289. 10.1038/nature04288 16251954

[B27] Panksepp J. (1998). *Affective Neuroscience.* New York, NY: Oxford University Press.

[B28] Pap G. (2021). A dream series and two methods of dream interpretation: a dream coding method according to U. Moser and I. V. Zeppelin in individual psychological practice [Eine Traumserie und zwei Methoden der Traumdeutung: Die Traumkodierungsmethode nach U. Moser und I. V. Zeppelin in der individualpsychologischen Praxis]. *Z. Freie Psychoanal. Fors. Individualpsychol.* 8 58–86. 10.15136/2021.8.1.58-86

[B29] Payne J. D. Nadel L. (2004). Sleep, dreams, and memory consolidation: the role of the stress hormone cortisol. *Learn Mem.* 11 671–678. 10.1101/lm.77104 15576884 PMC534695

[B30] Scarpelli S. Bartolacci C. D’Atri A. Gorgoni M. De Gennaro L. (2019). The functional role of dreaming in emotional processes. *Front. Psychol.* 10:459. 30930809 10.3389/fpsyg.2019.00459 PMC6428732

[B31] Siclari F. Valli K. Arnulf I. (2020). Dreams and nightmares in healthy adults and in patients with sleep and neurological disorders. *Lancet Neurol.* 19 849–859. 10.1016/s1474-4422(20)30275-1 32949545

[B32] Siegel J. M. (2001). The REM sleep-memory consolidation hypothesis. *Science* 294 1058–1063. 10.1126/science.1063049 11691984 PMC8760621

[B33] Stern D. N. (2020). *The Motherhood Constellation: A Unified View of Parent-Infant Psychotherapy.* Abingdon-on-Thames: Routledge.

[B34] Stickgold R. Hobson J. A. Fosse R. Fosse M. (2001). Sleep, learning, and dreams: off-line memory reprocessing. *Science* 294 1052–1057. 10.1126/science.1063530 11691983

[B35] Varvin S. Fischmann T. Jović V. Rosenbaum B. Hau S. (2012). Traumatische träume: streben nach beziehung. *Psyche* 66 937–967.

[B36] Zhao H. Li D. Li X. (2018). Relationship between dreaming and memory reconsolidation. *Brain Sci. Adv.* 4 118–130.

