# Supplementary material for: Dreams and Trauma Changes in the Manifest Dreams in Psychoanalytic Treatments – A Psychoanalytic Outcome Measure
Source: Front Psychol. 2021 Sep 14;12:678440. doi: 10.3389/fpsyg.2021.678440 (PMC8476791; doi:10.3389/fpsyg.2021.678440)
Supplement: Supplementary file 1 [file Data_Sheet_1.pdf]

Supplement: Comparing clinical and laboratory dreams with patients from the LAC Depression Study using the ZDPCS

As mentioned in our paper, in selected case studies of the LAC Study we looked at dreams patients reported in the clinical situation and compared them to dreams of the same time elicited at the sleep laboratory using the ZDPCS method of Moser & von Zeppelin (1996), to see whether we could discern changes of dream-atmosphere, relational capacities and problem-solving capabilities (i.e., self-agency). We were also interested to see if those changes occurred in both types of dreams – the laboratory dreams and the ones reported in the clinical situation.

The following steps were taken to prepare the reported dreams for analysis with the ZDPCS:

First the reported dreams were linguistically processed, i.e., they were given a verbal form that reflects the dreamt dream as well as arrange it in its sequential sequence. For this purpose, an identificatory attitude towards the dreamer is very helpful, in which one wanders through his dream world together with him, in order to be able to develop an immediate, present feeling for the events taking place. This should then also be reflected in the verbal formulations, which includes, for example, that the narration is set in the present tense. Peculiarities of the narrative style are omitted, as well as all explanations, ideas and comments which are communicated during the narration, however important they may be for an understanding. The goal of this specific dream analysis is to reconstruct the mental activity limited to the dream itself, but especially the pictorial-sensual sequence of the dream, which is normally hidden by the linguistic structure of the dream narration. The mental processes within a dream are taken into consideration on three different levels: 1. the sensual concrete level (mostly visually pictorial), which is assumed to be the basic and characteristic one for the dream; 2. the level of verbal relations, i.e., language spoken in the dream; 3. the level of cognitive processes in the form of thinking that takes place in the dreamer himself or attributed by the dreamer to an object of the dream.
Having put the dream into such a form, the next step is to segment it into individual situations. The transition from one situation to the next is defined by changes determined by the activity of a regulating mental entity, the 'dream organizer'. Such changes may be, for example, the appearance of new or the disappearance of existing cognitive elements (CE), changes in the form of interaction or a change in the level of mental activity. At these points, the dreamer focuses on something new that can initiate or cause a change in the dream event. Thereafter each situation is coded according to manual using the codes as explicated in table 3S (s. below).

For this purpose, all CEs and interactions appearing in the dream are objectively recorded and systematically represented according to the very differentiated, model-guided coding system. One may consider this coding system an operationalization of the dream work.
First, the elements of the positioning field (PF) are coded, which are substitutes of the dream-complex to be worked on, selected by the dream organization under the dominance of the security principle. They usually constitute a PLACE and sometimes a social setting that includes all positioned person processors. In the area of PLACE there are only distance relations. The cognitive elements (cognitive element [CE], subject processor [SP; usually the dreamer], object processor [OP]) may have specifying attributes (ATTR) on which future interactions hang as potentialities, where persons contain a greater potential than inanimate objects. Dream-internal affects are not yet present, but a certain affective moodiness may be

found as a diffuse perception of an affectivity condensed in PLACE and distributed over the whole positioning field.
After that, if present, the "loco-time motions" (LTM) are coded, i.e., spatial and temporal movements and trajectories in the positioning field, where also no interactive interactions exist yet. Hereby, the positioning field is changed, avoiding for the time being (or even finally) the development of interactive relations or making such a development more probable.
The interactive relations (IR) between cognitive elements finally characterize the processes in the interaction field (IAF). Interactive relations always cause changes in the current relation, be it in the form of a change of self or as a change of the relation of the subject processor with others or the relation between other objects. In addition, there are the explicit affective reactions (EX-AFF-R. i.e., "I was very afraid"). It is now possible to observe the affective relational regulation of the dreamer. These relations can be divided into resonant (IR. RES) and responsive (IR. RESP) relations. Resonant relations are those in which behavior, affects, motivations, desires, beliefs and so on are paralleled between the partners, which is done by way of unilateral or reciprocal affect induction. In responsive interactions, on the other hand, the behavior of the partners involved is not parallel but circular, and regulation is reciprocal. While resonant interactions aim at maintaining and securing a current relationship relation and thus have a stabilizing effect, responsive interactions aim at a real change of the same. Where it becomes clear, one can additionally make a distinction in both forms whether the subject feels more as an agent or as an object of the interaction, that is, whether the object is to be changed or whether one allows oneself to be changed by the object. The relationship between two objects can also be purely physical (IR.PHYS) or kinesthetic (IR.KIN), in which case the affective communication is ‘switched off’, but in the pre-conceptual animistic thinking of the dream, these also have an important meaning.

The patient discussed in our paper, was also willing to spend a night in the sleep-laboratory. Therefore, we could compare the following dream from the clinical situation, taken from the first 6 month of therapy, with a laboratory dream which may serve as an exemplary specimen here:

„I am in a narrow tunnel, kind of a tube. Behind me my brother is crawling. We cannot go backwards – behind us is the stormy sea. The tunnel becomes narrower and narrower. I wake up in panic. “

In comparison, his laboratory dream of the same week during therapy was blander although palpably full of anxiety and the feeling of being left alone helplessly:

“I am walking through a building – a residential building. I don’t know to which destination. Down the stairs – there are elevators. I walk through a door, behind it there are my parents, my brother. I try to talk to them. Then there is a fellow [female] student – her face is alienated. I am surprised and bewildered, happy to see her again. I ride elevators up or down. During the ride the floor of the elevator suddenly drops underneath me and is gone. I look outside – there are gigantic hangars with tools. They are deserted. I gaze for several minutes. There is an underground passageway. I am scared. Where does it go to? I am uncertain. A ride into the unknown?”

The Zurich Dream Process Coding System (ZDPCS) of the two dreams reveals interesting facts (see table 1S). Both dreams depict threatening situations: fear and panic are the dominant affects. The dream subject is in a helpless situation inundated with severe anxieties. No ‘helping Other’ is present in the dream. The dream from the clinical situation is shorter

and ends in open panic. The laboratory dream, though longer, has more interrupts – a sign for unbearable accumulation of affects, which must be interrupted – but has more distancing and failing interactions. The latter may be interpreted as a sign of the dreamers' lack of problem-solving capacities; he feels helplessly extradited to the situation he is in.

Dream from clinical situation					Laboratory Dream				
**Dream narrative**	**Sit**	**PF**	**LTM**	**IAF**	**Dream narrative**	**Sit**	**PF**	**LTM**	**IAF**
I am in a narrow tunnel, kind of a tube.	S1	SP (Dreamer)<br>PLACE (tunnel)<br>ATTR (narrow)<br>ATTR (tube)			*I am walking through a building – a residential building.*	S1	SP (Dreamer)<br>PLACE (building)<br>ATTR (residential)	LTM	
Behind me my brother is crawling.	S2	SP (Dreamer)<br>OP_1 BEK (brother)<br>POS REL	LTM		*I don't know to which destination.*	C.P.			
We cannot go backwards – behind us is the stormy sea.	S3	SP (Dreamer)<br>OP_1 BEK (brother)<br>PLACE (sea)<br>ATTR (stormy)<br>POS REL		IR.C RES LTM<br>FAIL (cannot go backwards)	*Down the stairs – there are elevators.*	S1	SP (Dreamer)<br>CEU (stairs)<br>CEU MULT (elevators)<br>POS REL		
The tunnel becomes narrower and narrower.	S4	SP (Dreamer)<br>PLACE (tunnel)<br>ATTR (narrow)		IR.D (IR.S)	*I walk through a door, behind it there are my parents, my brother.*	S2	SP (Dreamer)<br>CEU (door)<br>CEU (door)<br>POS REL<br>OP BEK (parents)<br>OP BEK (brother)	LTM	
I wake up in panic	EX AFF-R				*I try to talk to them.*	S3	SP (Dreamer)<br>OP BEK MULT (them)		IR.C int
					Then there is a fellow [female] student – her face is alienated. I am surprised and	S4	SP (Dreamer)<br>EX AFF R (surprised…)		

					bewildered, happy to see her again.		OP BEK (student)<br>ATTR<br>OP PART OF (face)<br>ATTR (alienated)		
					I ride elevators up or down.	S5	SP (Dreamer)<br>CEU (elevators)	LTM	
					During the ride the floor of the elevator suddenly drops underneath me and is gone.	S6	SP (Dreamer)<br>CEU (elevator)<br>CEU PART OF (floor)<br>POS REL		IR.D<br>(IR.S)
					I look outside – there are gigantic hangars with tools. They are deserted. I gaze for several minutes.	S7	SP (Dreamer)<br>CEU MULT (hangars)<br>ATTR (gigantic, deserted)	LTM<br>perc	
					There is an underground passageway.	S8	SP (Dreamer)<br>CEU (passageway)<br>ATTR (underground)		
					I am scared. Where does it go to? I am uncertain	EX<br>AFF<br>R			

Table 1S: ZDPCS dream coding comparing clinical dreams with laboratory dream of the first 6 months of psychoanalysis
(Sit: situation; PF: Positioning Field; LTM: Loco-time-motion; IAF: Interaction-Field. See Table 2S for further abbreviations)

As is discussed in detail in another paper the manifest dreams of this severely traumatized patient changed obviously during psychoanalysis (Leuzinger-Bohleber & Fischmann, 2018). In the frame of our paper, we can only refer to one other example of a clinical dream in the third year of psychoanalysis:

"I played with the famous jazz guitarist Ralf Towner. It went quite well, and it was fun. I didn't fail and the neck of the guitar was not soft[1] *(laughs). The guitarist played along with my improvisations and held back. Of course, I knew that he is better than me, but this did not matter – it was just great fun…"*

The corresponding laboratory dream from the same week, goes as follows:

"I was on the way with someone, whom I cannot name. He was familiar, but I cannot put a name to him. And we had a strange substance with us. In the beginning, it was a lump of earth or clay and he showed me how to make new forms out of it. In fact, not by processing it but by crumbling it. And this was interesting; so, he crumbled this thing, and it became fine flakes, fell and when it reached the ground new forms emerged. I tried to do it too but did not manage in the beginning – I thought it did not really work for me. But he said: "no, this is quite good already. It's not perfect yet, but I should keep on trying and it will get better, and it will work. And I still thought it didn't really work for me and that the product was baddish…"

The dreams from the third year of psychoanalysis reveal a very different picture in the ZDPCS which again, show similar topics and structures in the clinical as well as in the laboratory dream. In his dream from the clinical situation, he wakes from his dream highly affectively aroused (as in the dreams mentioned above), but this time he feels elated and during most of the dream he is in responsive interaction with an 'helping' object. The laboratory dream is in line with this insofar as that the dreamer is in a positively responsive interaction with a 'helping Other'. He does not have to be perfect: he is held and contained by a tolerant object which motivates him to continue his learning process.

As discussed in our paper: These dreams may have illustrated to a degree the way Mr. X.'s early traumatization become observable in his manifest dreams and how this changed during the treatment. The underlying traumatic complex that governed the dream organization at the beginning of treatment was successively better integrated in the psychic functioning of the patient. The dream coding showed how the dreamer established an increasing feeling of self-agency, control and basic trust in a helping other. From a methodological point of view, it is important that these transformations in the manifest dream content could be found in the clinical dreams as well as in the laboratory dreams.

[1] The patient refers to another "funny" dream. Before the dream he had a conflict with his wife which wasn't treated openly. Instead, the conflict led to an erectile dysfunction. Then he dreamed, that he played on a guitar which had a very soft neck…

**Dream from clinical situation**					**Laboratory Dream**				
**Dream narrative**	**Sit**	**PF**	**LTM**	**IAF**	**Dream narrative**	**Sit**	**PF**	**LTM**	**IAF**
I play with the famous jazz guitarist Ralf Towner. It goes quite well.	S1	SP (Dreamer)<br>OP BEK (Towner)<br>ATTR (famous)<br>ATTR (jazz g.)		IR. C RES	*I was on the way with someone, whom I cannot name. He was familiar.*	S1	SP (Dreamer)<br>OP (someone)<br>ATTR BEK		IR.C RES<br>LTM
it is fun	EX AFF R				*but I cannot put a name to him*	C.C.			
I don't fail and the neck of the guitar is not soft	S2	SP (Dreamer)<br>CEU (guitar)<br>PART OF (neck)<br>ATTR (not soft)			*we have a strange substance with us. In the beginning a lump of earth or clay*	S1	ATTR (have)<br>CEU (substance)<br>ATTR (strange, lump, like clay)		(s. above)
The guitarist plays along with my improvisations and holds back.	S3	SP (Dreamer)<br>OP BEK (guitarist)<br>ATTR (held back)		IR.C RES	*he shows me how to make new forms out of it. In fact, not by processing it but by crumbling it.*	S2	SP (Dreamer)<br>OP (he)<br>CEU (substance)<br>CEU mult (forms)		IR.C RES (shows)
Of course, I know that he is better than me, but this does not matter –	C.P.				*this is interesting*	C.C.			

it is just great fun….	EX AFF R				*he crumbles this thing, and it becomes fine flakes*	S3	SP (Dreamer) OP (he) CEU (thing) PART OF (flake) ATTR (fine)		IR.D (IR.C KIN (crumble) IR.D PHYS (become flakes)
					falls down	S4	SP (Dreamer) CEU (substance)	LTM	
					and when it reaches the ground new forms emerge	S5	SP (Dreamer) CEU (substance) CEU (forms) MULT ATTR (new)		IR.C PHYS (new emerge)
					I then also try to do it too but do not manage in the beginning	S6	SP (Dreamer)		IR. C KIN (try) FAIL
					I think it does not really work for me.	C.C.			
					he then says: "no, this is quite good already. It's not perfect yet, but keep on trying and it will get better and it will work.	S7	SP (Dreamer) OP (he)		V.R. (says)

Table 2S: ZDPCS dream coding comparing clinical dreams with laboratory dream of the third year of psychoanalysis
(Sit: situation; PF: Positioning Field; LTM: Loco-time-motion; IAF: Interaction-Field)

ATTR	attribute
ATTR BEK	attribute known
C.P.	explicit cognitive processes
CE	cognitive element
CEU	inanimate cognitive element
CEU MULT	multiple inanimate cognitive elements
CEU PART OF	part of an inanimate cognitive element
EX AFF-R	explicit affective reaction
IAF	Interaction-Field
IR.C	connectionistic interactive relation
IR.C INT	intentional IR.C
IR.C. KIN	IR.C – felt kinesthetically
IR.C KIN FAIL	failed IR.C – felt kinesthetically
IR.C PHYS	physical IR.C
IR.C RES	resonance relation
IR.C RESP	response relation
IR.D	displacement relation
IR.D PHYS	physical displacement relation
IR.S	interaction-relation-self
LTM	"loco-time-motion"
LTM FAIL	failed LTM
OP	object processor
OP BEK	known OP
OP BEK MULT	multiple, known OPs
OP PART OF	part of an OP
PLACE	place, location, site
PF	positioning field
POS REL	positioning relation
SIT	situation
SP	subject processor
V.R.	verbal relation
V.R. DIAL	dialogic V.R.
(())	Content of the secondary interactive field

Table 3S: Legend of the dream codes applied
